# Dataset of levels and masses of lipid species in healthy, asymptomatic and symptomatic leaves of *vitis vinifera* L. ‘Malvasia fina’ affected by ESCA complex disease

**DOI:** 10.1016/j.dib.2020.106469

**Published:** 2020-10-28

**Authors:** Piebiep Goufo, Isabel Cortez

**Affiliations:** a Centre for the Research and Technology of Agro-Environment and Biological Sciences, Universidade de Trás-os-Montes e Alto Douro, Quinta de Prados, 5000-801 Vila Real, Portugal; b Departamento de Agronomia, Universidade de Trás-os-Montes e Alto Douro, Quinta de Prados, 5000-801 Vila Real, Portugal

**Keywords:** Grapevine leaf stripe disease, ESCA, Lipidomics, Lipid maps classes, White rot, Brown wood streaking, Symptoms, Metabolomics

## Abstract

The dataset presented in this paper comprises the masses of 208 lipid species and other compounds of lipid metabolism, and their levels in leaves of vines with brown wood streaking and grapevine leaf stripe, two symptomatic expressions of Esca complex disease (ESCA). Healthy, asymptomatic and symptomatic leaves were collected from the cultivar Malvasia Fina grown in a vineyard. The lipidome of these leaves was characterized using a platform consisting of an Ultrahigh Performance Liquid Chromatography and a Gas chromatography equipment coupled to a Q-Exactive Hybrid Quadrupole-Orbitrap high resolution/accurate Mass Spectrometer interfaced with a heated electrospray ionization source. The analysis permitted the detection of 158 molecular species of known identity and 50 species of unknown structural identity. The area counts of these molecular species is reported in the dataset, along with fold changes (log2-ratio), *P*-values (Welch's two-sample *t*-test), and *q*-values (false discovery rate) from all pairwise comparisons among experimental groups. These statistical data are intended to serve as means of identification for lipid species whose levels were altered by the disease, and which could be used as biomarkers of symptom emergence and disease progression. Because of few studies on the subject, the association between modulation of lipid biosynthetic pathways and disease progression in grapevine has remained poorly understood. The analysis of the data described here has already provided new perspectives regarding the pathogenesis of ESCA leaf symptom formation. Reanalysis of these data would undoubtedly unravel some physiological roles played by lipids in the adaptation of vine plants to stressful conditions.

## Specifications Table

**Table:** 

Subject	Agricultural and Biological Sciences
Specific subject area	Plant pathology and Plant lipidomics
Type of data	Table Chart
How data were acquired	Automated MicroLab Liquid Handling System (Hamilton Robotics, Inc., Reno, NV, US) for lipid extraction; Ultrahigh Performance Liquid Chromatography (ACQUITY UPLC System, Waters Corporation, Milford, MA, US), Gas Chromatography (Shimadzu GC-2010-Plus, Kyoto, Japan), and Q-Exactive Hybrid Quadrupole-Orbitrap high resolution/accurate mass spectrometer (HRaMS) interfaced with a heated electrospray ionization (HESI-II) source (Thermo Fisher Scientific, Waltham, MA, US) for separation of lipids; Xcalibur QuanBrowser 3.0 (Thermo Fisher Scientific, Waltham, MA, US) for peak integration; Metabolon's Laboratory Information Management System (LIMS, Metabolon, Inc, Morrisville, NC, US) for peak identification.
Data format	Raw Analyzed
Parameters for data collection	Leaf samples were collected from the grapevine cultivar Malvasia Fina, which was naturally infected in the vineyard with ESCA-related pathogens. Annual inspections during a period of 6 years allowed to distinguish the vines, and to collect (*n* = 6) leaves with similar water contents from unaffected (healthy) and wood-affected (asymptomatic) vines and leaves with varying degrees of damage (chlorosis and scorching/spotting) from affected (symptomatic) vines.
Description of data collection	A workflow for extraction, separation and identification of lipid species from lyophilized powdered leaves was established based on a multiplexed MS-based platform. The platform is based on a couple of extraction steps resulting in five aliquots that are subjected to: (i) three UPLC separations using reverse phase chromatography optimized for hydrophilic, hydrophobic and basic compounds, (ii) one UPLC separation using hydrophilic interaction liquid chromatography optimized for polar compounds, and (iii) one GC separation using capillary chromatography optimized for free fatty acids. Peak annotation was achieved using the LIMS library of ca. 10,000 MS/MS spectra of standard compounds.
Data source location	Institution: Universidade de Trás-os-Montes e Alto Douro City/Town/Region: Quinta de Nossa Senhora de Lourdes, Vila Real Country: Portugal Latitude and longitude (and GPS coordinates, if possible) for collected samples/data: 41 ° 17.12′ 31′’ N, 7 ° 44.07′ 22′’ W, 465 m
Data accessibility	Repository name: MENDELEY DATA Data identification number: 10.17632/4k49sk6s2w.3 (Lipidome of leaves of esca-affected grapevine) Direct URL to data: https://data.mendeley.com/datasets/4k49sk6s2w/3
Related research article	P. Goufo, I. Cortez, A Lipidomic analysis of leaves of esca-affected grapevine suggests a role for galactolipids in the defense response and appearance of foliar symptoms. Biology 9 (2020) 268. https://doi.org/10.3390/biology9090268

## Value of the Data

• A direct use of these data would be for identification of metabolite markers associated with delay appearance of ESCA-foliar symptoms in grapevine. Fold change and *t*-test data have been provided that should help in that respect. Additional analyses such as principal component, hierarchical clustering, and random forest could be performed to explore whether different classification methods nominate different candidate predictors of the presence of the disease.
• This type of data is likely to be extremely beneficial to researchers working on finding molecular markers that could assist in the current diagnostic approaches for ESCA. The identified biomarkers could be used by investors for the development and commercialization of a non-invasive biochemical method for early esca diagnosis by winegrowers.
• So far, no characterization has been made of global lipid changes that occur following infection of vines by pathogens. The lipidome data generated from the leaves of grapevine and reported in this paper could be used to determine whether and how reprogramming of lipid metabolism exert a role on the etiopathogenesis of ESCA and other vine diseases.
• These lipidomic data can also be analyzed together with other published grapevine-related OMIC data in order to discover new insights into the host's mechanisms of resistance, or to elucidate key issues in grapevine cell biology such as evolution and crop enhancement.
• Through pathway enrichment or correlation network analyses, data reported here could yield important information about the metabolic pathways associated with reduced foliar symptom expression that might consent to engineer resistant cultivars.

## 1 Data Description

**Fig. 1:**
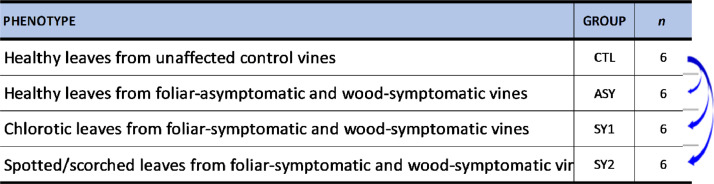
Study design: statistical comparisons contrasted leaves from each of the infected groups to those of the control uninfected group.

The data provided in the MENDELEY repository is related to the levels of lipid species in four experimental leaf groups (CTL, ASY, SY1, and SY2) as described in Fig. 1**.** There were six biological replicates for each of the leaf group, for a total of 24 samples coded PD-UDTD-00001 to PD-UDTD-00024 (**Table S1**).

In total, 208 lipid species and other compounds of lipid metabolism were successfully detected in all 24 replicate samples and are listed along with their retention indexes and mass-to-charge ratios (*m/z*) in **Table S1**; each compound is assigned a unique ID (001 to 208). Chromatographic peaks corresponding to lipid species were quantified as area-under-the-curve detector ion counts and area counts were normalized to the sample weight to obtain the raw data in **Table S1**. The proportions of samples with successful quantification (detection rates) are also shown in the table. For each compound, the chromatographic condition used for quantification is listed i.e. LC/MS Pos Early, LC/MS Pos Late, LC/MS Polar, LC/MS Neg or GC/MS. Empirical names (LIPID NAME) and chemical structures (SMILES = Simplified Molecular-Input Line-Entry System) were generated for each given accurate mass. Compounds with the same mass and retention time that could not be integrated independently (i.e. isobars) are denoted by adding a number sign after the isobaric species name and a number within brackets after its abbreviation. Peaks that had reproducible retention time, mass, and MS/MS spectra, but could not be associated with a chemical library are given a numerical designation starting with X (e.g. X–23440); these are labeled “UNNAMED” in the column “TYPE”. The designation enables these compounds to be documented in future experiments and to be identified by future acquisition of a matching purified standard or by classical structural analyses.

**Fig. 2:**
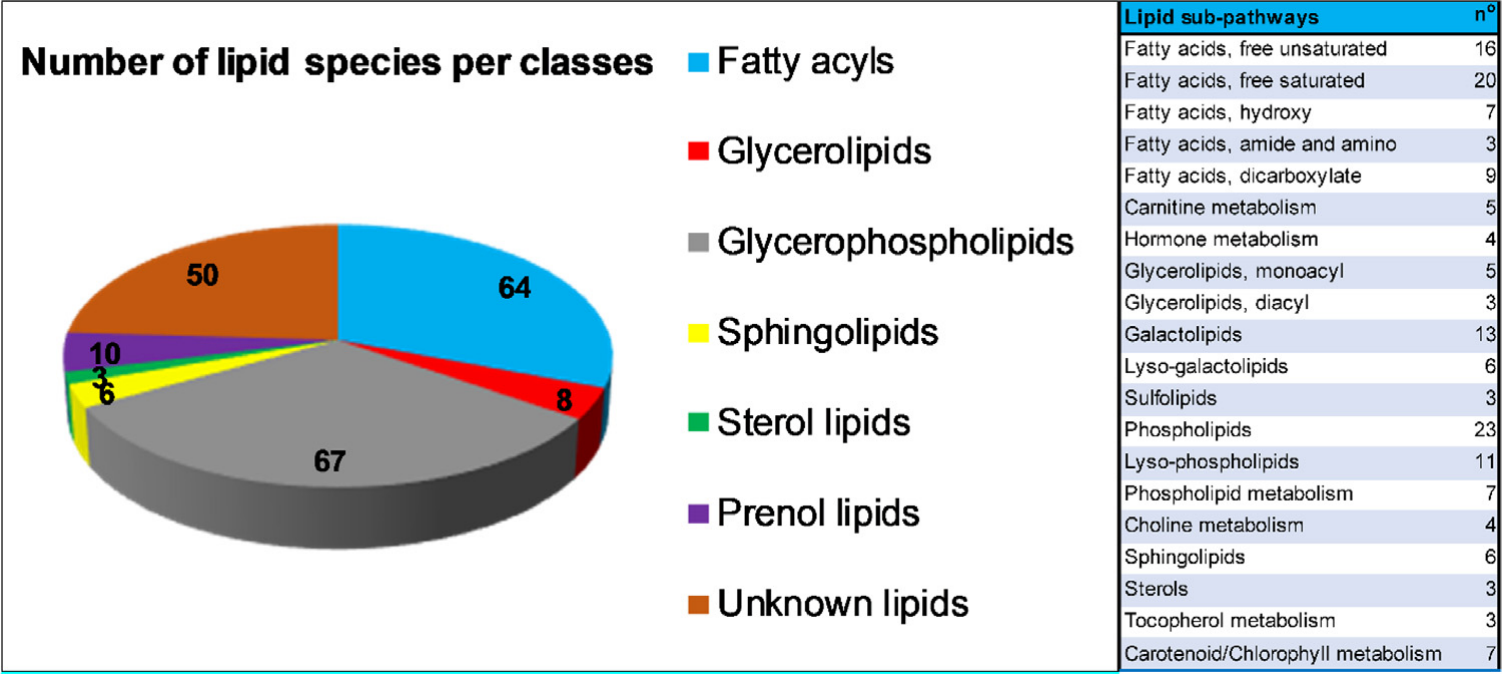
Molecular species were classified according to Lipid Metabolites and Pathways Strategy (LIPID MAPS, https://www.lipidmaps.org/), and Metabolon Pathway orders (Metabolync™, https://www.metabolon.com/).

Identified compounds represented all major Lipid Metabolites and Pathways Strategy (LIPID MAPS, https://www.lipidmaps.org/) classes, including fatty acyls, glycerolipids, glycerophospholipids, sphingolipids, sterol lipids, and prenol lipids (Fig. 2). Compounds are listed in the dataset according to Metabolon Pathway orders (Metabolync™, https://www.metabolon.com/); this corresponds to 20 lipid sub-pathways (Fig. 2). Whenever possible, these compounds are linked to public databases that contain MS/MS spectra, namely Kyoto Encyclopedia of Genes and Genomes (KEGG, https://www.genome.jp/kegg/), Human Metabolome Database (HMDB, http://www.hmdb.ca/), Plant Metabolic Pathway Databases (PLANT CYC, https://plantcyc.org/). For several molecular species, ID codes for other databases of chemical molecules (COMP/In-house, CHEM, CAS, PUBCHEM, CHEMSPIDER) are provided.

**Fig. 3:**
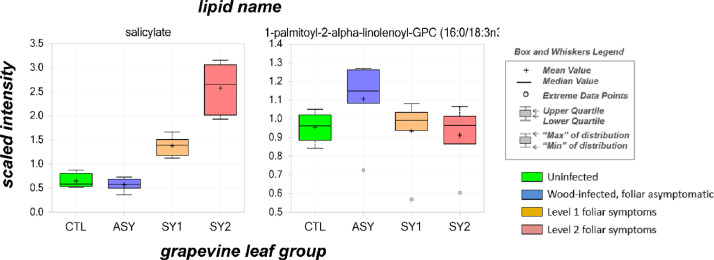
Examples of box-and-whisker plot graphical displays for visualizing level changes in molecular species among experimental leaf group. This figure is partly adapted from [1].

Scaled imputed data used in statistical analyses are reported [1]; these data were obtained by rescaling the raw area count for each molecular species to set the median equal to 1. As provided in **Table S2**, fold changes (log2 ratios) and *P*-values (Welch's two-sample *t*-tests) were applied to screen for potential differences between the means. An estimate of the false discovery rate (*q*-value) is also provided to take into account the multiple comparisons. The following colors are used for highlighting differences between the groups: white = *P* > 0.10 for unaffected levels, dark green = *P* ≤ 0.05 for decreased levels, light green = *P* ≤ 0.10 for decreased levels, red = *P* ≤ 0.05 for increased levels, and light red = *P* ≤ 0.10 for increased levels. Box-and-whisker plots for 208 molecular species are sorted by biochemical pathway in **Figure S1;** 29 of the plots are also present in [1]. An example of box-and-whisker plot is provided in Fig. 3. Readers can refer to **Table S3** for a general definition of colors and further descriptions of codes used in the dataset. For ease of presentation, abbreviations are provided for all molecular species names in the tables and charts.

## 2 Experimental Design, Materials and Methods

### 2.1 Field work

#### 2.1.1 Characteristics of the study site

Samples used for lipidomic analyses were collected in a vineyard naturally infected by ESCA-associated pathogens. The vineyard, called Quinta de Nossa Senhora de Lourdes (465 m, 41 ° 17.12′ 31′’ N, 7 ° 44.07′ 22′’ W), is in the city of Vila Real in Portugal. In the immediate surroundings of the vineyard are three other vineyards with different cultivars (north, west and east) separated from each other by roads, and a cherry field (south). The vineyard was planted on an area of 2700 m^2^ in 1995 with *V. vinifera* L. ‘Malvasia Fina’ grafted on 19-17-Castel rootstock. Malvasia Fina, also known as Boal, is a white grape cultivar grown historically in Portugal. Initially, the vineyard had 1247 vines organized in 22 rows. With time, some vines have been uprooted for studies and some have died; missing vines have not been replanted. The distance between the rows is 1.80 m for 1.12 m spacing between vines in the row. Different training systems are used for canopy management. Based on observations of leaf symptoms during the last decade, ESCA is the main disease in the vineyard [2]. However, the main cause of vine death is suspected to be Armillaria root rot.

In the sampling year, vines were grown in the field at an average annual air temperature of 13.97  °C with 75% relative humidity and a 16-h photoperiod (1350 μmol/m^2^/s). Precipitation was limited with 880.7 mm average rainfall and the vineyard had no irrigation system. The soil (Anthrosol) was uniform across the experimental plot and was characterized by 62% sand, 25% silt, and 13% clay (pH 4.2). Pruning, fertilization and plant protection measures were undertaken according to Plant care guidelines developed by Universidade de Trás-os-Montes e Alto Douro.

#### 2.1.2 Definition of experimental groups

The sampling protocol was developed using four rows of vines (a block of 243 vines) facing north and trained to a bilateral cordon according to the royal-type trellis system. An inspection period of six continuous years gave sufficient time to identify all the vines showing internal and external signs of ESCA in the study block. Visual inspection of all individual vines was conducted yearly after grape cluster harvesting, following published recommendations [3,^4^,5]. Notations were made based on all external symptoms i.e., apoplexy on shoots, tiger stripes on leaves, black measles on berries and unproductive vines (dead or missing). The occurrence of internal symptoms was evaluated using woods cores (ca. 5 mm of diameter and 60–100 mm long) extracted with an increment borer from the trunk (ca. 20 cm above the soil surface and 10 cm below the head). Before hand-drilling the trunk, the increment borer was sterilized by dipping it in a 25% sodium hypochlorite solution for 1 min, followed by rinsing with 70% ethanol and sterile distilled water. The aspect and volume of necrotic wood was assessed visually [6].

Affected vines were characterized as having diverse shapes and colors of wood and leaf deterioration and necrosis, and were grouped into I, II, III, and IV. Group I comprised of healthy unaffected control vines (CTL) that had minor or non-necrotic wood tissues and did not express foliar symptoms in any of the inspection year. Group II comprised of wood-symptomatic and foliar-asymptomatic vines (ASY) that expressed tiger stripe-like foliar symptoms no more than three times during the 6-year survey period. These vines exhibited clear signs of brown wood streaking, which is a wood deterioration characteristic of ESCA [7,8]. Group III comprised of wood-symptomatic and foliar-symptomatic vines (SY1) with brown wood streaking that have continuously expressed tiger stripe-like foliar symptoms. Leaves collected from these vines were mostly chlorotic: light-green areas developing between the veins and at the margin of the leaves that eventually turned rust- or reddish-colored throughout the growing season. Group IV also comprised of wood-symptomatic and foliar-symptomatic vines (SY2) with brown wood streaking that have continuously expressed tiger stripe-like foliar symptoms. However, from these vines were collected spotted and scorched leaves: rounded or irregular spots between the veins or along the leaf margins which usually spread outward to the distal part of the shoot, coalesced, and finally became necrotic. It is known that these chlorotic (SY1) and spotted/scorched (SY2) symptoms will assume a tiger stripe pattern at later stages of the disease progression [2]. Symptomatic leaves were sampled on vines that first showed symptoms the same week. This was made possible by recording the appearance and progression of foliar symptoms three times per week in June, and once per week in July, August and September.

#### 2.1.3 Collection of leaf samples

For each of the experimental groups, ten vines were identified. Approx. 10 leaves facing and opposite a grape bunch were collected from the central portion of each vine, immediately frozen with liquid nitrogen and stored at −80  °C. A similar set of samples was kept at normal temperature and dried in an oven at 85  °C to a constant weight for water content determination. Once the water content was known, samples from six vines (representing independent biological replicates with similar water contents ca., 65%) were selected for lipidomic analyses. Prior to these analyses, samples were freeze-dried and pulverized using a blender (Electrodomesticos Taurus, Aromatic Ver-II, Barcelona, Spain).

### 2.2 Laboratory work

#### 2.2.1 Chemicals used for extraction and separation of lipids

All organic solvents and reagents of analytical grade (≥ 98%) were purchased from Sigma-Aldrich (St. Louis, MO, US) or Panreac Química SLU (Barcelona, Spain). Chemicals for lipid extraction included methanol, chloroform, potassium chloride, hexane, 14% boron trifluoride, and anhydrous sodium sulfate. Standard mixtures of fatty acid methyl esters (FAME37), alkanes mixtures, LC-MS grade acetonitrile and methanol were also purchased from Sigma-Aldrich, as well as ammonium bicarbonate, ammonium formate, ammonium hydroxide, perfluoropentanoic acid and formic acid. Water was obtained from a Millipore Ultra-high purity water dispenser (Billerica, MA, US). Isotopically labelled compounds (e.g., methyl-d3 stearic acid and d7-β-sitosterol) were purchased from C/D/N Isotopes (Pointe-Claire, Quebec, Canada) and Santa Cruz Biotechnology (Dallas, TX, US).

#### 2.2.2 Extraction of lipids from leaf samples

Lipids were extracted using a modification of the protocol proposed by [9]. The protocol describes two extractions steps that yield five extract fractions for separation by chromatography. In the first extraction step, 20 mg sample was added with 400 µL methanol. The components were mixed thoroughly by homogenizing (GenoGrinder 2000, Glen Mills Inc, Clifton, NJ, US) for 2 min at room temperature. Following centrifugation (Pro-Analytical CR400R, Centurion Scientific Ltd, Chichester, UK) at 1500 × *g* for 5 min (25  °C), the top organic phase was collected and divided into four fractions. The fractions were transferred to a speed vacuum concentrator (Zymark Corporation's Turbovap, Hopkinton, MA, US) to remove the organic solvent and the resulting residues frozen at −80  °C. In the second extraction step, 50 mg sample was added with a mixture of methanol, chloroform, water, and potassium chloride as described in [2]. The resulting extract was transesterified with 14% boron trifluoride in methanol [10] to obtain a residue (fifth fraction) that was kept at −80  °C. All liquid handling steps were performed using the Hamilton Robotics (Reno, NV, US) MicroLab STAR® system.

#### 2.2.3 Ultrahigh performance liquid chromatography and gas chromatography conditions

The fractions were removed from the freezer and stored under a nitrogen stream at room temperature for ca. 15 h before being reconstituted in solvents compatible to each of the five methods used for chromatographic separations.

Fraction 1 was reconstituted in 50 µL 0.1% formic acid in water and analyzed by UPLC using acidic positive ion conditions chromatographically optimized for the elution of hydrophilic compounds (LC/MS Pos early). The extract was gradient-eluted from a C18 column (BEH—C18, 2.1 × 100 mm, 1.7 µm particle size; Waters Corporation, Milford, MA, US) at 40  °C and 0.35 mL/min using (A) 0.1% formic acid and 0.05% perfluoropentanoic acid in water and (B) 0.1% formic acid and 0.05% perfluoropentanoic acid in methanol (0%B to 70%B in 4 min, 70–98%B in 0.5 min, and 98%B for 0.9 min).

Fraction 2 was also reconstituted in 50 µL 0.1% formic acid in water and analyzed by UPLC using acidic positive ion conditions, but chromatographically optimized for the elution of hydrophobic compounds (LC/MS Pos late). The extract was gradient-eluted from the aforementioned C18 column at an overall higher organic content using (A) 0.01% formic acid in water and (B) 0.05% perfluoropentanoic acid in 60/40 methanol/acetonitrile; and the same gradient profile as for Fraction 1.

Fraction 3 was reconstituted in 50 µL of 6.5 mM ammonium bicarbonate in water (pH 8.0) and analyzed by UPLC using basic negative ion optimized conditions (LC/MS Neg). The basic extract was gradient-eluted from a separate base-dedicated C18 column using (A) 6.5 mM ammonium bicarbonate in water (pH 8) and (B) 6.5 mM ammonium bicarbonate in 95/5 methanol/water (same gradient profile as above).

Fraction 4 was reconstituted in 50 µL ammonium formate (pH 10.8) in water and analyzed via negative ionization following elution at 0.35 mL/min from a Hydrophilic Interaction Liquid Chromatographic (HILIC) column (BEH Amide, 2.1 × 150 mm, 1.7 µm inner diameter, Waters Corporation, Milford, MA, US) using a gradient consisting of (A) 10 mM ammonium formate in 15% water, 5% methanol, 80% acetonitrile; pH 10.8 and (B) 10 mM ammonium formate in 50% water, 50% acetonitrile; pH 10.6 (0%B for 4 min, 0–50%B in 2 min, 50–80%B in 5 min, 80–100%B in 1 min, and 100%B for 2 min). The pH of all solvents was adjusted by adding ammonium hydroxide, and the conditions were optimized for targeting polar compounds (LC/MS Polar).

Fraction 5 was reconstituted in 1 mL hexane. Following vortexing for 3 min, the upper phase was analyzed by GC, with elution on a DB-225MS column (30 m long × 250 μm i.d. × 0.25 μm thick film; Agilent, Wilmington, DE, US). Separation conditions targeted free fatty acids (GC/MS) and were as follows: split ratio of 5:1; inlet temperature of 270  °C; carrier gas, helium (1 mL/min flow rate, 39 cm/s linear velocity); oven temperature, 200  °C (5 min followed by 5  °C/min till 220  °C, and then 5 min); ion source temperature, 230  °C; interface temperature, 270  °C; ionization voltage, 70 eV.

Reversed phase separation of molecular species was achieved by Ultrahigh Performance Liquid Chromatography using an ACQUITY UPLC System (Waters Corporation, Milford, MA, US). Capillary gas chromatography was with a Shimadzu GC-2010 Plus (Kyoto, Japan). The machines were coupled to a Q-Exactive Hybrid Quadrupole-Orbitrap high resolution/accurate mass spectrometer (HRaMS) interfaced with a heated electrospray ionization (HESI-II) source (Thermo Fisher Scientific, Waltham, MA, US). In all cases, an aliquot of 5 μL of extract fraction was loaded in the chromatograph using an automatic injector and analyzed using MS.

#### 2.2.4 Mass spectrometry method

Samples were analyzed in positive and negative ion modes depending on the fractions. The MS interface capillary was maintained at 350  °C and the corona discharge current was set at 5 μA. The spray voltages for the positive and negative ion injections were 4.50 and 3.75 kV, respectively. Nebulization was with nitrogen, with a sheath gas flow of 40 (arbitrary units) and an auxiliary gas flow of 5 (arbitrary units) for both positive and negative injections. The nebulizer temperature was set at 400  °C.

The mass spectra were recorded alternately between full-scan (MS) and all-ion fragmentation-scan (MS^n^) modes, and over the period between 1 and 15 min within UPLC gradients and GC. The scan range varied slightly depending on the fraction analyzed, but covers approximately 70–1000 *m/z*. The resolution for all scans was set at 35,000 (measured at 200 *m/z)* and approximately 9 scans were performed per second, which restricted the loading time to 100 ms. MS/MS normalized collision energy was set to 40, activation Q 0.25, and activation time 30 ms, with a 3 *m/z* isolation window. MS/MS scans were collected using dynamic exclusion with an exclusion time of 3.5 s. The system was calibrated internally as needed to maintain a mass error < 5 ppm for all internal standards (IS) monitored.

#### 2.2.5 Assessment of data quality

Before extraction, each powdered sample was added with a cocktail (15 μL of 100 μg/mL) of six recovery standards in methanol (d6-abscisic acid, DL-2-fluorophenylglycine, methyl-d3 stearic acid, tridecanoic acid, 4-chlorophenylalanine and d7-β-sitosterol or d6-cholesterol), which permitted the monitoring of extraction efficiency. The percent recovery was ≥ 95% for each standard.

All reconstituted fractions were spiked with a series of isotopically labeled IS at fixed concentrations before injection into the MS. The following ISs were used to assess instrument performance and to serve as retention time (RT) markers for chromatographic alignment: d7-glucose, d3-methionine, d3-leucine, d8-phenylalanine, d5-tryptophan, Cl-phenylalanine, Br-phenylalanine, d15-octanoic acid, d19-decanoic acid, d27-tetradecanoic acid, d35-octadecanoic acid for UPLC and alkanes mixtures C8-C40 (even) for GC.

Technical replicate samples generated by combining a small portion of each of the 24 experimental samples were also analyzed. Water blanks and solvent blanks were used to provide a baseline reference signal and segregate external contamination sources, respectively. Experimental samples were randomized across the platform run, with technical replicates and blanks spaced evenly among the injections.

Instrument variability was determined by calculating the median relative standard deviation (RSD) for the ISs. Overall process coefficient of variation was determined by calculating the median RSD for all endogenous lipids present in technical replicate samples. Median RSD for instrument and process variability were 7% and 8% (< 10%) respectively, indicating that all aspects of the analysis process were operating within specifications.

#### 2.2.6 Data extraction and processing

Data acquisition was performed using Xcalibur QuanBrowser 3.0 and Lab Solution 5.71 for UPLC and GC, respectively. Various user-defined peak threshold values were included for baseline correction, chemical noise subtraction, chromatogram alignment, peak detection, and integration. This included a signal-to-noise ratio > 5, peak area > 50,000 (LC) and 100 (GC), peak width = 0.2 min. After this preprocessing step, raw data were extracted and a list of peaks passing above threshold criteria was obtained. These peaks were organized by mass-to-charge ratio (*m/z*) within a matrix along with their respective intensities for each sample.

#### 2.2.7 Identification and quantification of lipids

Peak detection was carried out using an in-house developed data management system, namely the Metabolon's Laboratory Information Management System (LIMS). Using retention indexes (RI), LIMS was first used to perform a RT correction of the output matrix based on RT markers placed throughout the chromatographic time window. Then, the resulting MS/MS data were searched against the LIMS library of purified standards and routinely detected unknown entities by comparing their specific *m/z*, fragment ion spectra, and RIs within user-given ranges. The LIMS chemical library is generated from ca., 10,000 MS/MS spectra of authenticated standard compounds, including their associated adducts, in-source fragments, and multimers.

Compound identifications were based on three criteria: (i) RI within a narrow window of the proposed identification, typically 75 RI units of the proposed identification or approximately 5 s, (ii) accurate mass match to the library of +/− 10 ppm, (iii) MS/MS forward and reverse scores above 80%. Identifications were automatically approved if all the above criteria were met [9,11]. Only one method (LC/MS Pos Early, LC/MS Pos Late, LC/MS Neg, LC/MS Polar, or GC/MS) was chosen to represent compounds redundantly detected during the injections. When isomers belonging to the same isobaric species were found they were denoted by adding a number within brackets after the isobaric species name.

Hundreds of molecular species were successfully detected in all samples. Compounds that were detected in at least 33% of all replicates for the same test group were included in subsequent analyses. Peaks were quantified as area-under-the-curve detector ion counts and normalized to the sample weight. The raw area count for each compound was rescaled through the division of each sample value by the median value for this specific compound to obtain the scaled imputed data.

#### 2.2.8 Fold change calculations, *t*-test analyses and box-and-whisker plot visualizations

Statistical analyses consisted of a series of pairwise comparisons among experimental groups (CTL, ASY, SY1, SY2) using the Excel add-in application “Statistical Analysis Tool” (http://prime.psc.riken.jp/compms/others/main.html#Statistics). Following imputation of missing values with the minimum observed value for each compound, fold changes were calculated as log2 ratio of the scaled imputed mean of an experimental group and that of another experimental group. Welch's two-sample *t*-tests were used to compare the means of the different leaf groups. Before these analyses, scaled imputed data were log10-transformed to normalize distributions. A *P*-value correction was performed by estimating the false discovery rate − which was obtained by the *q*-value −, thereby accounting for multiple comparisons. For a better visualization of the data, box-and-whisker plots of means were constructed for each molecular species.

## CRediT Author Statement

**Piebiep Goufo:** Conceptualization, methodology, validation, formal analysis, investigation, data curation, writing– original draft preparation, writing– review and editing, visualization. **Isabel Cortez** Conceptualization, methodology, validation, resources, writing– review and editing, supervision, project administration, funding acquisition.

## Ethics Statement

This article does not contain any studies involving animals or humans performed by any of the authors

## Declaration of Competing Interest

The authors declare that they have no known competing financial interests or personal relationships which have, or could be perceived to have, influenced the work reported in this article.

## References

[1] Goufo P., Cortez I. (2020). A lipidomic analysis of leaves of ESCA-affected grapevine suggests a role for galactolipids in the defense response and appearance of foliar symptoms. Biology (Basel).

[2] Goufo P., Marques C.A., Cortez I. (2019). Exhibition of local but not systemic induced phenolic defenses in Vitis vinifera L. affected by brown wood streaking, grapevine leaf stripe, and apoplexy (ESCA complex). Plants.

[3] Calzarano F., Osti F., D'agostino V., Pepe A., Della Pelle F., M De rosso, Flamini R., Di Marco S. (2017). Levels of phytoalexins in vine leaves with different degrees of grapevine leaf stripe disease symptoms (ESCA complex of diseases). Phytopathol. Mediterr..

[4] Lecomte P., Diarra B., Carbonneau A., Rey P., Chevrier C. (2018). ESCA of grapevine and training practices in France: results of a 10-year survey. Phytopathol. Mediterr..

[5] Sofia J., Mota M., Gonçalves T.M., Rego C. (2018). Response of four Portuguese grapevine cultivars to infection by Phaeomoniella chlamydospora. Phytopathol. Mediterr..

[6] Del Frari G., Gobbi A., Aggerbeck M.R., Oliveira H., Hansen L.H., Ferreira R.B. (2019). Characterization of the wood mycobiome of Vitis vinifera in a vineyard affected by ESCA. Spatial distribution of fungal communities and their putative relation with leaf symptoms. Front. Plant Sci..

[7] Claverie M., Notaro M., Fontaine F., Wery J. (2020). Current knowledge on Grapevine trunk diseases with complex etiology: a systemic approach. Phytopathol. Mediterr..

[8] M Magnin-Robert, Adrian M., Trouvelot S., Spagnolo A., Jacquens L., Letousey P., Rabenoelina F., Harir M., Roullier-Gall C., Clément C., Schmitt-Kopplin P., Vallat A., Abou-Mansour E., Fontaine F. (2017). Alterations in grapevine leaf metabolism occur prior to ESCA apoplexy appearance. Mol. Plant-Microbe Interact..

[9] Evans A.M., DeHaven C.D., Barrett T., Mitchell M., Milgram E E. (2009). Integrated, nontargeted ultrahigh performance liquid chromatography/electrospray ionization tandem mass spectrometry platform for the identification and relative quantification of the small-molecule complement of biological systems. Anal. Chem..

[10] Goufo P., Ferreira L.M.M., Carranca C., Rosa E.A.S., Trindade H. (2014). Effect of elevated carbon dioxide concentration on rice quality: proximate composition, dietary fibres and free sugars. Cereal Chem..

[11] Goufo P., Singh R.K., Cortez I. (2020). A Reference list of phenolic compounds (including stilbenes) in grapevine (Vitis vinifera L.) roots, woods, canes, stems, and leaves. Antioxidants.

